# The counting principle makes number words unique

**DOI:** 10.1515/cllt-2023-0105

**Published:** 2024-03-29

**Authors:** Mira Ariel, Natalia Levshina

**Affiliations:** Department of Linguistics, Tel Aviv University, Tel Aviv, Israel; Neurobiology of language, 10187 Max Planck Institute for Psycholinguistics, Nijmegen, Netherlands; Centre for Language Studies, Radboud University, Nijmegen, Netherlands

**Keywords:** numerals, numeric expressions, distributional semantics, word2vec, prototypes, lexical domains

## Abstract

Following Ariel (2021. Why it’s hard to construct ad hoc number concepts. In Caterina Mauri, Ilaria Fiorentini, & Eugenio Goria (eds.), *Building categories in interaction: Linguistic resources at work*, 439–462. Amsterdam: John Benjamins), we argue that number words manifest distinct distributional patterns from open-class lexical items. When modified, open-class words typically take selectors (as in *kinda table*), which select a subset of their potential denotations (e.g., “nonprototypical table”). They are typically *not* modified by loosening operators (e.g., *approximately*), since even if bare, typical lexemes can broaden their interpretation (e.g., *table* referring to a rock used as a table). Number words, on the other hand, have a single, precise meaning and denotation and cannot take a selector, which would need to select a subset of their (single) denotation (??*kinda seven*). However, they are often overtly broadened (*approximately seven*), creating a range of values around *N*. First, we extend Ariel’s empirical examination to the larger COCA and to Hebrew (HeTenTen). Second, we propose that open-class and number words belong to sparse versus dense lexical domains, respectively, because the former exhibit prototypicality effects, but the latter do not. Third, we further support the contrast between sparse and dense domains by reference to: synchronic *word2vec* models of sparse and dense lexemes, which testify to their differential distributions, numeral use in noncounting communities, and different renewal rates for the two lexical types.

## 1 Introduction

To date, many of the claims about the meaning and interpretation of number words have been based on intuitions, whereby linguists (or experimental participants) judge an expression’s interpretation in a leisurely manner, reasoning about the state of affairs behind the utterance, so that they can safely judge the utterance as true or false (Cummins 2015; Krifka 2002). To borrow Kahneman’s 2011 terminology, this is a relatively slow and conscious – System 2 – type of processing. Although no one can deny that such processes play a role in human cognition, we believe that linguists should focus more on the *default* interpretations of linguistic expressions, those which result from the fast, automatic interpretative processes characteristic of Kahneman’s System 1, because these shape language use and, consequently, language structure. We can identify such automatic interpretations, which are predicted by the speakers to be the default for their addressees, by analyzing the distributional patterns of numeral modifiers, following the famous principle “You shall know a word by the company it keeps” (Firth 1957: 11). The relevant patterns here considered mainly pertain to the different category modifiers favored by number words versus open-class lexemes.

What, then, are the default interpretations associated with numerals and with typical open-class lexemes? We propose that numerals are rigidly interpreted as precise numerosities (as ‘exactly N’). This sets numerals apart from the flexible typical lexemes. Typical lexemes are vague, which is why the speaker-intended concept they evoke is constructed ad hoc. While this construction is based on the lexical core, this core often undergoes significant contextual adjustments (Carston 2002). What enables the flexibility of typical lexemes is the prototype effects associated with their meanings (Lakoff 1987; Langacker 1987). The two features of interest to us here are having: (i) multiple members – denotations, some of which may be more central (prototypical) than others, and (ii) flexible boundaries around them, which relatively easily pave the way for potentially blurring the distinction between category and noncategory denotations.1Note that just like Armstrong et al. (1983) and van Tiel et al. (2021), we do not assume that manifesting prototype effects necessarily entails that concepts and/or linguistic meanings lack boundaries altogether. We also do not assume that the distinction between central and peripheral members (exemplars) is necessarily based only on shared features. Family resemblance and analogy play a role too. Central and peripheral exemplars of some linguistic item may very well be based on salience and frequency of use. Finally, central and peripheral denotations may vary by context. These two features enable open-class items to serve as a means for evoking a rather large number of ad hoc concepts, without recourse to a conventional one-to-one relation between words and concepts (e.g., *cut* is differently interpreted with *hair* or *cake* or *grass*, etc., as its complements).2One of our referees would like us to make do with only two analytic terms: abstract semantic meanings and contextually determined referents based on these meanings. Contra the referee, however, we think that (i) exemplars, which may have different levels of entrenchment, are stored in the speakers’ minds, and that (ii) ad hoc concepts are contextually created, such that (iii) specific referents are picked on their basis. These ad hoc concepts modify the more entrenched exemplars stored for the lexeme. The distribution of *kinda* testifies to the relevance of these assumptions. Numerals, on the other hand, do not exhibit prototype effects. Their borders are not flexible, and they typically do not evoke contextually different ad hoc concepts (the interpretation of *seven* remains ʻexactly seven’ whether it modifies *hairs* or *cakes*).

**Figure 1:**
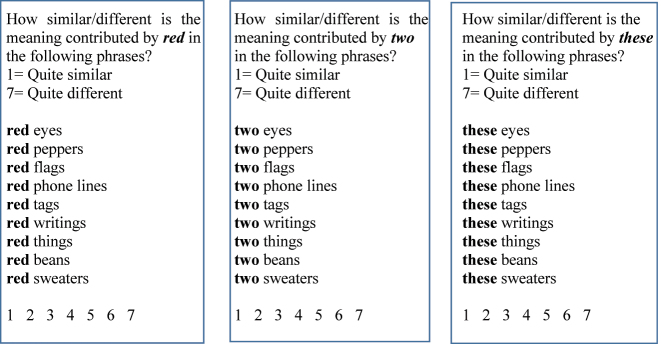
Examples of an adjective, a numeral, and a determiner preceding the same set of plural nouns.

Before we turn to our main topic, namely the distributional differences between typical lexemes and numerals, let us first see that the two are indeed interpreted differently. Here is one illustration for the difference. In an informal expert judgment questionnaire, sixteen linguists were asked to determine how similar/different the meaning contributed by three types of nominal modifiers was when modifying different nouns. A high grade on a 7-point scale indicated different interpretative contributions by the targets, and a low grade indicated a similar contribution. The modifiers tested were numerals (*two, five, eight*), adjectives (*red, straight, empty*), and determiners (*these, the, some*). Nine naturally occurring tokens of the adjectival expressions, when immediately preceding a count noun, were extracted from the Santa Barbara Corpus of Spoken American English (SBC, and when necessary, from The Longman Corpus of Spoken American English, LSAC), and three triplets were created. In each, the target expressions (an adjective, some numeral, or some determiner) preceded the same set of plural count nouns, in order to see if and how much the target word interpretation varied according to the noun it modified. One such triplet is displayed in Figure 1 (the other two, as well as the raw results, can be found in Supplementary Materials SM1).3All supplementary materials can be found at: https://doi.org/10.17605/OSF.IO/AQEKT.


The results were very clear. The meaning contributed by the tokens of *red*, *empty*, and *straight* was rather different in the different contexts (average similarity/difference grade: 4.8), but the tokens of each of the three numerals made quite similar meaning contributions (average similarity/difference grade: 1.04). Indeed, in this respect, the numerals pattern with the highly restricted set of grammatical determiners (average grade: 1.4),4While *these* and *the* actually received a similar similarity grade as the numerals (1.2), *some* was judged as more variant (1.9), probably because it is also a quantifier. despite the fact that the former carry conceptual, rather than procedural content. Unlike adjectives, which denote properties the objects are associated with, numerosity just isn’t an inherent property of most objects (see Culbertson et al. [2020] about the rather weak association between numbers and the objects they quantify). The result is that the interpretation of numerals tends to be invariant, faithful to its literal meaning. Indeed, it is “stubbornly” precise.

The contrast between the following pair of examples is another illustration of the differential interpretations associated with numerals versus typical lexemes:

1.

a.

**Table:** 

Kato Keilin (about O.J. Simpson): He was **upset but he wasn’t upset** (cited by Carston 2002: 324).

b.

**Table:** 

?? ∼They were **seven but not seven**.^5^

5 ∼ indicates a nonattested example.

As an open-class lexeme, *upset* can be differently contextually adjusted on its two occurrences (‘somewhat upset’ versus ‘significantly upset’). This is why (1a) is not contradictory. (1b) is contradictory because the numeral is interpreted as ‘seven’ on both occurrences.

Contextual adjustments involve narrowing and/or broadening (Carston 2002). Consider narrowing first. As mentioned above, the meaning of typical lexemes admits a wide range of multiple denotations and manifests prototype effects.6When speaking about prototypes, we mean prototypicality effects apparent in *language use* and in *lexical* entries. We do not make claims about how categories are represented in the mind – as abstract prototypes, exemplars, or in any other possible way (Murphy 2004). However, the existence of prototypicality effects in language use – for example, in the form of gradient goodness of exemplar membership, or fuzzy boundaries – is undeniable (Bybee 2006; Geeraerts 2010). As will be seen below, modifiers such as *kinda* support this assumption. But the speaker-intended concepts are often narrower, allowing only a specific subset of the potential denotations (see [1a] again). Naturally, different contexts call for differently narrowed concepts:

2.

a.

**Table:** 

ALINA: … (H) otherwise I’d have no **hair** left. (SBC: 006)

b.

**Table:** 

There wasn’t any **hair** growing (LSAC)

The ‘hair’ in (2a) refers to the prototypical, human head hair, but the ‘hair’ in (2b) refers to the body hair of a seal, who was branded and hence had no hair where it was branded.

Broadening, or loosening, occurs when the nonrigid boundaries surrounding lexical meanings are pushed away, allowing the consideration of meanings, which are relatively close to the category concept (relative to the specific context), despite the fact that they do not in themselves fall within the lexical meaning.7Broadening and narrowing are not mutually exclusive. More often than not they are part of a single process of contextual adaptation (Wilson and Carston 2007). However, this is irrelevant for interpretation of our empirical results. This process is very common in metaphorical uses (e.g., *he’s such* ***a baby***, LSAC), but it is not restricted to such uses. The ‘hair’ in (3) stands for a broadened hair concept, chicks’ down feathers:

3.

**Table:** 

AMY: … they were … losing all of their … **hair**. (SBC: 039)

Consider numerals now. Unlike typical lexemes (e.g., *hair*, *nice*), (i) bare numerals (e.g., *six*) cannot at all undergo narrowing, because they are associated with single, rather than multiple denotations. The distributional implication from their inability to narrow is that they cannot be modified by denotation selectors such as *real*(*ly*) (which selects specifically prototypical denotations) or *kinda* (which selects nonprototypical denotations). (ii) Bare numerals are typically not broadened either, because the borders surrounding them are rigid, so they are routinely interpreted precisely. In order to be associated with an approximate value (e.g., ‘about seven’), the numeral is preferably explicitly marked for approximation (e.g., *about seven*). In such cases, of course, it’s not the numeral *per se*, but rather, the overt approximator, or broadener, that (compositionally) gives rise to the approximate interpretation. This is exactly what we find in our study of COCA and HeTenTen (Section 2): numerals occur with approximators (broadeners), but hardly ever with selectors.

We present two types of distributional evidence in support of our claim about the difference between typical and numerical expressions. The first is based on the idea that speakers use specific category modifiers (e.g., *kind of, roughly*) to guide addressees on how to interpret the modified item in the specific context. Such modification is especially useful when the speaker wishes to block the default interpretation associated with the given item. Following Ariel (2021), we predict that since numeral expressions and typical lexemes are each associated with different default interpretations, they should favor different category modifiers. Specifically, the differences pertain to narrowing and broadening category modifiers. The flexible typical lexemes prefer selectors, while the rigid numerals prefer broadeners (Section 2).

We then explain that these differences in distributional patterns stem from the fact that the numerals belong to a dense semantic domain, which is why their meaning is not subject to prototype effects.8Note that the density relevant here is *not* the assumption that there’s an infinite number of *numbers* between any two (noninteger) numbers. See Section 3.1 for a definition of dense and sparse lexical domains. The meanings of typical lexemes, on the other hand, belong to sparse lexical domains, which is why they are subject to prototype effects (Section 3.1).

Our second distributional study, a *word2vec* analysis (Section 3.2), is a wholistic examination of the distributional patterns of numerals and typical lexemes. This analysis too shows a remarkable difference between the two lexical systems.

We note that our results can shed light on an ongoing debate about the meaning of number words. Some linguists assume an under-specified meaning (Carston 1998; Geurts 2006), while others adhere to the classical analysis with a lower-bound-only interval meaning for the numerals (‘at least *N’*) (see *inter alia*, Chierchia and McConnell-Ginet 1990; Horn 1989).9Robyn Carston (p.c.) too now subscribes to the precise analysis. On these approaches, the precise interpretation of numerals results from a postlexical process of strengthening by scalar implicature (“no more than *N*”). Our empirical results support an ‘exactly *N’* *encoded* analysis for the numerals (Ariel 2006; Breheny 2008; Bultinck 2005; Kadmon 2001; Koenig 1991). Indeed, we show that it is nonprecise interpretations that are routinely derived with the help of overt markers (approximators), in which case it is the modifiers that compositionally contribute the interval readings. Nonetheless, all we argue for here is that based on their distributional patterns, exact, rather than approximate *readings*, are the default for numerals.

## 2 Modified numerals: extending the empirical basis

The goal of this section is to further support the claim originally made in Ariel (2021) that the meaning of numerals is not subject to prototype effects. Ariel’s original claim, based on different distributions regarding category modifiers for numerals versus typical lexemes, was supported with limited English corpus data from The Santa Barbara Corpus of Spoken American English – SBC (Du Bois et al. 2000–2005). This is why we set out to extend its empirical basis. We present parallel findings for the much larger COCA (Corpus of Contemporary English) (Section 2.1) and for a rather different language, Hebrew (based on the HeTenTen corpus, Section 2.2). Just like SBC, these corpora show that numerals are much more frequently overtly broadened (e.g., by *more or less*) than typical lexemes. Selectors (among multiple denotations, e.g., *kinda*), on the other hand, are attested for typical lexemes, but virtually never for numerals.

Before we present our data, however, we need to defend our decision to treat all number expressions as single lexemes in a single lexical domain, despite the fact that some of them are multi-word expressions, such as *twenty-seven* or *two thousand and eleven*. We take it that there is no controversy about the psychological reality of a *single* number line, which combines (and intersperses) mono-lexeme and multi-lexeme number expressions. In fact, what’s monolexemic in one language (e.g., Hebrew *mataim* ‘two hundred’) is multi-lexemic in other languages, such as English.10Note that Bauer and Huddleston (2002]: 1716) consider numerals up to 100 single word expressions, although some numbers are coded by compounds (e.g., *twenty-one*). It’s quite clear that the more frequent the number the more compact its coding, starting with monomorphemic forms (1–12 in English), mono-lexemic forms (the teens and the power of ten numbers). Our point is that for our purposes, all numerals are lexeme-like. Surely, there’s no difference in the conceptual functioning of these morphologically different numeral types. Within a single language too, the relation between say, *nineteen* and *twenty* (two monolexemes), is no different from that between *twenty-one* and *twenty-two* (two complex numerals), nor from *twenty* and *twenty-one* (one mono-lexemic, the other multi-lexemic). We, therefore, suggest that the sparsity/density of compositional expressions is inherited from their lexical components.

In fact, there’s some evidence that at least some of the multi-word numerals do function similarly to single lexemes. This claim is not only based on conceptual facts but also on formal facts. First, just like mono-lexical number expressions, the multi-lexeme expressions each denote a *single concept*: It is the meaning of *fifty seven* or *ten thousand and three* (‘57’, ‘10,003’), as a unit, that is *invariant* across different contexts, different communities, and different periods of time. This is typically not true for (sparse) multi-word phrases.

Moreover, some of these multi-lexeme expressions are formally mono-lexemic-like in that they lack overt markers of compositionality. ‘1007’ can be pronounced one thousand *and* seven, reflecting full compositionally, or one thousand seven, which is partially opaque. No wonder then that the components of some multi-lexeme numerals show some loss of independence. For example, English *three thousand* “should have been” *three thousand* ***s*** (Bauer and Huddleston 2002: 1717); the Hebrew counterpart of, e.g., *three thousand* shows ‘three’ in construct state (*shloshet*, rather than *shlosha*), which cannot occur independently. In fact, multi-lexeme numbers are typically fixed (portmanteau) complex forms, just like words are, even though compositionality would have allowed variation. Idiosyncrasies abound (Comrie 2022). For example, the only way to express ‘399’ in Supyire (numeral base 80) is with the counterpart of *Eighty four and twenty three and ten and five four*, which must be understood as: 80 × 4 + 20 × 3 +_i_10 +_ii_ five + _iii_ 4. In principle, there could be a number of compositional alternatives to express this number. Note, moreover, that multiplication is zero-marked here, whereas addition is overtly marked for (i) and (ii), but not for (iii).

Finally, it’s not surprising that at least some mono-lexemic expressions are the diachronic product of compositional phrases (e.g., *fifty* = *five ten*). Hebrew *axat.esre* ‘eleven’, for example, literally ‘one ten’ is showing signs of fusion, as it is often pronounced as a single phonological word *xacre*. Since these complex numbers are lexeme-like conceptually, and even formally sometimes, we refer to the whole number line as a dense *lexical* domain so as to underscore its different patterning from sparse-domain lexemes.11But readers who remain unconvinced can treat the number line as a semantic domain, rather than as specifically a lexical domain.


### 2.1 COCA-modified numerals

We collected all cardinal numerals from COCA and determined how many times a numeric expression occurred with a broadener or with a selector. The corpus, the process of data extraction, and the list of modifiers are detailed in the Supplementary Materials (SM3).

The total frequencies of numeric expressions and their frequencies when broadened and selected from in different segments of COCA are displayed in Table 1.

**Table 1:** Frequencies of numeric expressions in COCA (500 million tokens), extracted automatically.

	Spoken	Academic	Fiction	Newspapers	Magazines
All cardinal numerals	831,266 (100 %)	2,041,593 (100 %)	659,525 (100 %)	2,304,587 (100 %)	1,729,443 (100 %)
Modified by broadener	100,811 (12.1 %)	101,465 (5 %)	39,811 (6 %)	144,939 (6.2 %)	161,897 (9.4 %)
Modified by selector	1,116 (0.13 %)	1,093 (0.05 %)	405 (0.06 %)	629 (0.03 %)	872 (0.05 %)

The numbers show that the most informative registers (news and academic texts) contain the highest number of numeric expressions (the top row, see also Biber [1995]; Woodin et al. [2023]). The second and third rows show that the vast majority of numeric expressions are neither broadened nor selected from by the modifiers here checked for. Yet, they are much more often broadened than they undergo selection by the operators here examined (133.4 times more across the four genres). As much as 12.1 % of the spoken segment numerals are overtly broadened by one of the broadeners we checked (as compared with 0.13 % selections, 90.3 times more). This is still quite a lower rate of broadening than that found by Ariel (2021) – close to 25 %, but first, Ariel’s count was manual and took into account *any* broadener encountered. Moreover, while the COCA spoken data represent diverse transcribed TV and radio broadcasts, many of these may in fact be read out loud. Ariel’s data, on the other hand, were restricted to spontaneous face-to-face conversations (the most natural habitat for language use and change). Numerals are broadened the least frequently in the academic texts (5 %). This is not surprising because precision is crucial in academic discourse.

As for selectors, they occur very infrequently (with less than 0.2 % of all numeric expressions) in all segments. Moreover, if we take a closer look at the examples, we can see that very many of them are in fact spurious hits. Some of them modify nouns, not numerals, as in (4a) and (4b), from the spoken segment:

4.

a.

**Table:** 

We are not bashing **any one** group…

b.

**Table:** 

How are the Ten Commandments, the **real Ten** Commandments are doing in Hollywood…

To conclude, the large-scale data from COCA support our hypothesis: numeric expressions are much more often overtly broadened and virtually never undergo selection. This supports the idea that numerals do not comprise of multiple denotations.

### 2.2 HeTenTen-modified numerals

We used HeTenTen, a web-based corpus compiled using a web-crawler, for extracting the relevant Hebrew data.12We thank Ziv Peleg-Plotnik for the HeTenTen corpus searches, as well as for coding the data, later discussed with the first author. The details about the corpus and data extraction are provided in the Supplementary Materials (SM4). While the COCA searches were made on numeral expressions, which we then coded for whether they were modified by a number of category operators (broadeners and selectors), the Hebrew searches were made on modified numerals. In addition, we here also compare the modification of numerals with that of a typical lexeme. Three Hebrew denotation selectors were chosen: *Mamash* ‘real(ly)’, *dey* ‘pretty (much)’, and *sug shel* ‘kind of’.Each presupposes that multiple denotations are associated with the modified phrase and points to a different selection.13
*Mamash* selects a central category member (to the exclusion of peripheral members), and *dey* and *sug shel* select peripheral members (to the exclusion of central members). The difference between *dey* and *sug shel* is irrelevant to our examination.


We contrasted these selectors with one approximator, *be.erex* ‘approximately/more or less’, which can modify both numerals and typical lexemes. For comparison with the numerals, we chose an open-class adjective *rek* ‘empty’, which is an absolute (precise) adjective. As can be seen below, the fact that it stands for an extreme value does not mean it needs to be overtly broadened. Quite the opposite. We predicted that if modified, this open-class lexeme would mostly be modified by one of the selectors, rather than by the approximator. The numerals, on the other hand, were predicted to mostly be modified by the approximator, rather than by any one of the selectors.

Our raw results are displayed in Table 2.

**Table 2:** HeTenTen selectors and approximator with numerals and *rek* ‘empty’.

Modifier type/frequency	Selectors (*mamash, dey, sug shel*)	Broadener (*be.erex*)	Total
General operator frequency	888,513 **90.3 %**	95,756 9.7 %	984,269 100 %
Modified *rek* ‘empty’	185 **98.9 %**	2 1.1 %	187 100 %
Modified numerals	265 5.6 %	4,466 **94.4 %**	4,731 100 %

Percentages in bold stand for the predominant modification pattern.

Table 2 shows that an overwhelming majority of the operators we examined were selectors (over 90 %). *Be.erex* ‘approximately’ tokens only account for under 10 % of our operators.14These numbers only serve as a baseline for our comparison and cannot testify to the relative proportions of selectors versus approximators in general. Now, there were 5,503,344 expressions tagged as numerals in HeTenTen, and only 24,455 *rek* ‘empty’ tokens. In other words, there were 225 numerals per each token of *rek.* Nonetheless, there were only 1.4 times more selectors for the numerals than for the single typical lexeme. Although *rek* tokens were rarely modified by selectors (0.76 %, 1 in every 154 tokens), the parallel result for the numerals is significantly lower (0.005 %, 1 in every 20,767 tokens) – 157 times less.

The picture is reversed for the broadener we checked for. Virtually no tokens of *rek* ‘empty’ were overtly broadened (2, 0.008 %, or 1 in every 12,227 tokens), as compared with the numerals (4,466, 0.08 %, or 1 in every 1232 tokens). This means that proportionately, there are about 10 broadeners with numerals per one broadener for the typical lexeme. Examining the third and fourth rows of Table 2, we see that while the typical lexeme is overwhelmingly modified by the selectors, numerals were overwhelmingly modified by the broadener.

These findings are similar to the findings for English in Ariel (2021), as well as the COCA data presented in Section 2.1. But now, we must ask, how come a minority, albeit of a marginal size, of the numerals in English (4,115 cases in COCA) and Hebrew (265 cases) were seemingly modified by selectors. We manually examined all the Hebrew cases and found that these cases were for the most part only apparent exceptions. As many as 187/265 cases (70.6 %) were actually irrelevant for the question under discussion, for various reasons: search errors, cases where the number word does not in fact function as a numeral, cases where the “selector” has an altogether different function, or cases where the selector preceding the numeral actually modifies a phrase preceding it, or the selection pertains to the modified nominal rather than to the numeral (as in [4b] above).

Summing up, HeTenTen portrays the same picture as COCA, whereby numerals do not undergo selection. We are left with 78 cases of selectors modifying numerals (out of 4,731 modifiers), which represents a meager 1.6 % of modified numerals (the rest are broadeners).15The remaining 78 cases are analyzed in Ariel and Levshina (2021).
Section 3 presents our theoretical account for the differential distributions. But we must first argue against potential objections to our analysis (Section 2.3).

### 2.3 Countering potential objections

An apparent counter-example to our claim, which might be particularly salient to linguists, is the seeming naturalness of *exactly* as a numeral modifier. *Exactly N* is a very frequent collocation in linguistic analyses. But if, as we claim, numerals are precise by default, they should preferably collocate with the approximating *about* rather than with the narrowing *exactly*. Indeed, this is the case. A 200 random sample of *exactly* in BNC revealed that only 4 (2 %) are numerals. In comparison, an examination of a random sample of 500 *about* tokens revealed 79 cases where a numeral was modified by *about* (15.8 %). An exhaustive search for *exactly three/3* revealed 23 tokens, 0.023 % of all *three/3* tokens (100,279), but the 13,888 *about three/3* tokens constitute 1.4 % of *three/3* (600 times more). Similarly, *exactly fifty/50* yielded 6 tokens, 0.04 % of the total *fifty/50* tokens (15,851), but the 584 tokens of *about fifty/50* constitute 3.7 % of *fifty/50* (97 times more). *Exactly* rather collocates with open-class expressions, such as *the same* (1308/10,188, 12.8 % of the *exactly* tokens) and *the opposite* (66/10,188, 0.65 %). Indeed, 2.3 % of *the same* tokens (1308/57,487) are modified by *exactly*, as are 2.7 % of *the opposite* tokens (66/2459).

Another potential criticism might be that not all numerals are alike. Specifically, that while nonround numerals may indeed be interpreted precisely by default, this is not so for round numerals, nor for numerals within measurement phrases (e.g., *seven inches*) – the latter are assumed to be round too. Our claim is that round and measurement numerals too are precise by default, despite the fact that they are more easily taken as *compatible* with approximate values.16See Ariel (2004) for the distinction between interpretation and truth compatibility. This point is explicitly argued for in Katzir et al. (2024). We here note, however, that our findings above about the default preciseness of numerals do not result from the fact that we analyzed all numerals, where, one might think, nonround numerals are the majority. Based on a random sample of 120 number expressions in HeTenTen, we estimate that as many as 45.6 % of all numeral expressions are round and/or measurement numerals.

## 3 Dense versus sparse lexical domains

We take the findings from spoken American English in Ariel (2021) and the new findings for the large English and Hebrew corpora in Section 2 to have established that numerals can broaden with the help of overt broadeners, but they cannot undergo selection, even with overt selectors. In this respect, numerals contrast sharply with open-class lexemes. The goal of Section 3 is to explain why it is that numerals do not manifest the prototype effects shown by typical lexemes. We define dense and sparse domains in Section 3.1 and classify numerals as dense-system members and typical lexemes as sparse-system members. Section 3.2 presents a *word2vec* analysis as further evidence for the difference between the dense numeral domain and three sparse-domain typical lexemes.

### 3.1 Defining dense and sparse domains

Ever since Trier (1934), it is commonly assumed that words belong to different conceptual fields, each lexical item naming some parceled out conceptual subdomain of the field. Analyzing words encoding types of knowledge in Old and Middle High German, Trier proposed that lexemes offer a continuous coverage of the whole relevant conceptual field. We agree, however, with later researchers who disputed the full coverage idea (Lehrer 1974; Lyons 1977; Ullmann 1967). Indeed, there can be nonlexicalized regions within many conceptual domains. In addition, we assume that functionally relevant domains are not restricted to predefined *stable* conceptual fields, each falling under a single superordinate category. *Nice*, *attractive*, and *kind*, for example, belong to a single lexical domain in some contexts, but of course, they each participate in other lexical domains as well.

We define a lexical domain as a set of contextually determined paradigmatic lexical alternatives, where the different lexemes encode distinct, yet similar enough and even interdependent meanings. This entails that they potentially compete with each other in the same context. On our account, Trier’s definition of a lexical field is actually inappropriate for typical lexemes: Typical lexemes, we claim, form part of sparse lexical domains. However, his mosaic-style definition is just right for the conceptual domain of counting: The natural number expressions belong to a densely packed lexical domain (see again Note 8). It is the +1 successor principle that is responsible for the numerals’ membership in a dense lexical domain. We note that the number line it creates is reinforced by the high cultural salience assigned to the counting routine, which is recited by very young (Western) children even before they understand the meaning of the numerals (Wynn 1992). In fact, Casati (2014) already proposed that numerals (and calendrical lexemes) are nonstandard lexemes just because they are inseparable from what he takes to be a tacit morphological, representation of the number line.

Table 3 summarizes the differences between dense and sparse lexical domains.

**Table 3:** Sparse and dense lexical domains.

	Sparse lexical domain (e.g., ‘nice’, ‘table’)	Dense lexical domain (e.g., ‘six’)
**(1) Distinctness** between neighboring items	Large (on multiple parameters)	Minimal (on a single parameter)
**(2) Semantic space** between neighboring items	Partial coverage, a large “no man’s” (=nonlexicalized) land	Full coverage, no “no man’s” (=nonlexicalized) land
**(3) Boundaries** around items	Flexible, adjustable	Sharp, rigid
**(4) Meaning**	Context-sensitive, polysemous, open to change	Context-insensitive, monosemous, Resistant to change

Consider *six* and its immediate neighbors.

(1) While *six* denotes a distinct concept from both *five* and *seven*, the difference between them is minimal, as compared with the difference between, e.g., *nice* and *attractive* or *table* and *chair*. The difference between neighboring typical lexemes is neither minimal nor consistent.17There may in addition be a partial overlap between open-class lexemes (e.g., *boil* and *scald*). But the difference between adjacent numerals is defined over a single parameter (the +1 successor principle). Interestingly, the uniform (+1) difference between adjacent numerals is overlaid on top of a conserved cognitive perception of magnitude, where magnitude differences are not uniform. Thus, Dehaene’s (2011) participants perceived the difference between small numbers (e.g., *7* and *8*) as larger than that between larger numbers (e.g., *27* and *28*). The successor principle behind the natural number line renders these relative differences irrelevant, however, because they are not relevant to counting.
(2) All words’ meanings (and interpretations) are constrained by their neighbors. But for typical words, there need not be full coverage of the conceptual domain, and there is a rather large nonlexicalized conceptual gap between neighbors (e.g., between *nice* and *attractive*), which a typical word can then “encroach” on. This accounts for the context-adaptability of typical lexemes (recall *nice* interpreted as ‘close to attractive’ in [3]). There is no conceptual gap between two neighboring (counting) numerals.18Note that fractions and decimals do not participate in the default *counting* number line. This question is addressed in Ariel and Levshina (2021). This accounts for their rigid faithfulness to what we take to be their literal, precise meaning.
(3) We have already seen that the numerals’ boundaries are rigid. This is why it takes an explicit broadener to create an approximate numeral concept, where it is then the operator, rather than the numeral, that is responsible for the broadening. Typical lexemes, on the other hand, broaden quite freely in the absence of overt broadeners (see again [3]). The result is that numerals’ interpretations are rigidly precise, regardless of the context they’re embedded in. Typical lexemes, on the other hand, adapt to the specific context they occur in and create different ad hoc concepts (see again Section 1). Finally, (4) the synchronic invariance of interpretation associated with numerals is not conducive to potential diachronic meaning changes. But the multiple-denotation typical lexemes give rise to contextually adapted interpretations and are then conducive to meaning change. We cite evidence for the extremely low rate of diachronic change for the numerals in Section 4.3.

A note is here in order about round and measurement numerals, which seem to refute our universal preciseness assumption. We address this issue in Ariel and Levshina (2021), where we argue that while the default number line for *all* numerals is the dense, +1 number line, some numerals (measurement ones, as well as what we call Personality numerals) can *in addition* shift to a *sparse* numeral line, where they too can “encroach” on their neighbors.19Personality numerals are ones where the numeral carries an additional meaning on top of its numerosity, which is why it is not only minimally distinct from its dense domain neighbors (parameter 1 above). This is the case of round numerals (they form a collective unit), as well as, e.g., Biblical Hebrew *seven* (a magic number). At the same time, we argue that for these numerals too the default number line is the dense +1 domain.

### 3.2 A word2vec analysis

In this subsection, we use a *word2vec* model trained on COCA for comparison of numerals with a variety of typical lexemes. Our data show remarkably different distributional patterns for the two types of expressions. The model indeed shows that the number domain is highly densely populated, so that lexemes’ meanings are rather similar to each other, whereas typical lexemes form part of sparse domains, where meanings are relatively different from one another.20See SM5 for a similar Wordnet picture, based on a feature analysis.


In order to measure how dense different lexical fields are, and compare them with numerals, we used a vector space model. The model creates a vector space with hundreds of dimensions, as a rule. Every vector corresponds to a word. If two words are used in similar contexts, they will have comparable values on the same dimensions and will be located close to each other in the multidimensional semantic space (see an introduction of vector space models for usage-based linguists in Levshina and Heylen [2014]).

Specifically, we use a popular algorithm *word2vec* (Mikolov et al. 2013). This is a neural network that learns to predict words from their neighbors based on a large corpus. For example, how likely is the word *crown* to appear if some word in its neighborhood is *queen*? In order to find out, the algorithm builds two layers. One is the hidden layer, in which the rows are the context words, and the columns are hundreds of dimensions. Every word is represented by a vector with different values on those dimensions. The other one is the output layer, which is used to generate the probabilities of different words in the neighborhood of the context words. If two words are used in similar contexts, like *king* and *queen*, then *word2ve*c should have similar probabilities for other words in their neighborhood. For example, words like *crown* or *monarchy* would have similarly high probabilities given *king* or *queen*, while words like *microscope* and *proton* would probably have similarly low probabilities. An important idea is that in order to have similar output probabilities, the word vectors in the hidden layer should also be similar. So the words that predict other words in a similar way should have similar semantics and similar distributional vectors. For technical details, see SM6 in the Supplementary Materials.

The numerals we compare represent numbers from two to ninety-nine, which we compared with typical lexemes belonging to three different lexical domains: adjectives describing intelligence, precision adjectives, and verbs of cooking from Lehrer (1974). The complete lists of lexemes are available in SM6.

After we created the semantic vectors for all these words, we computed cosine measures, which represent semantic distances between the words. We calculated the average distances between the members of the fields, which helped us estimate how dense or sparse a lexical field is on average. We also used the distances to visualize the data in ordinal Multidimensional Scaling, as shown below.

**Figure 2:**
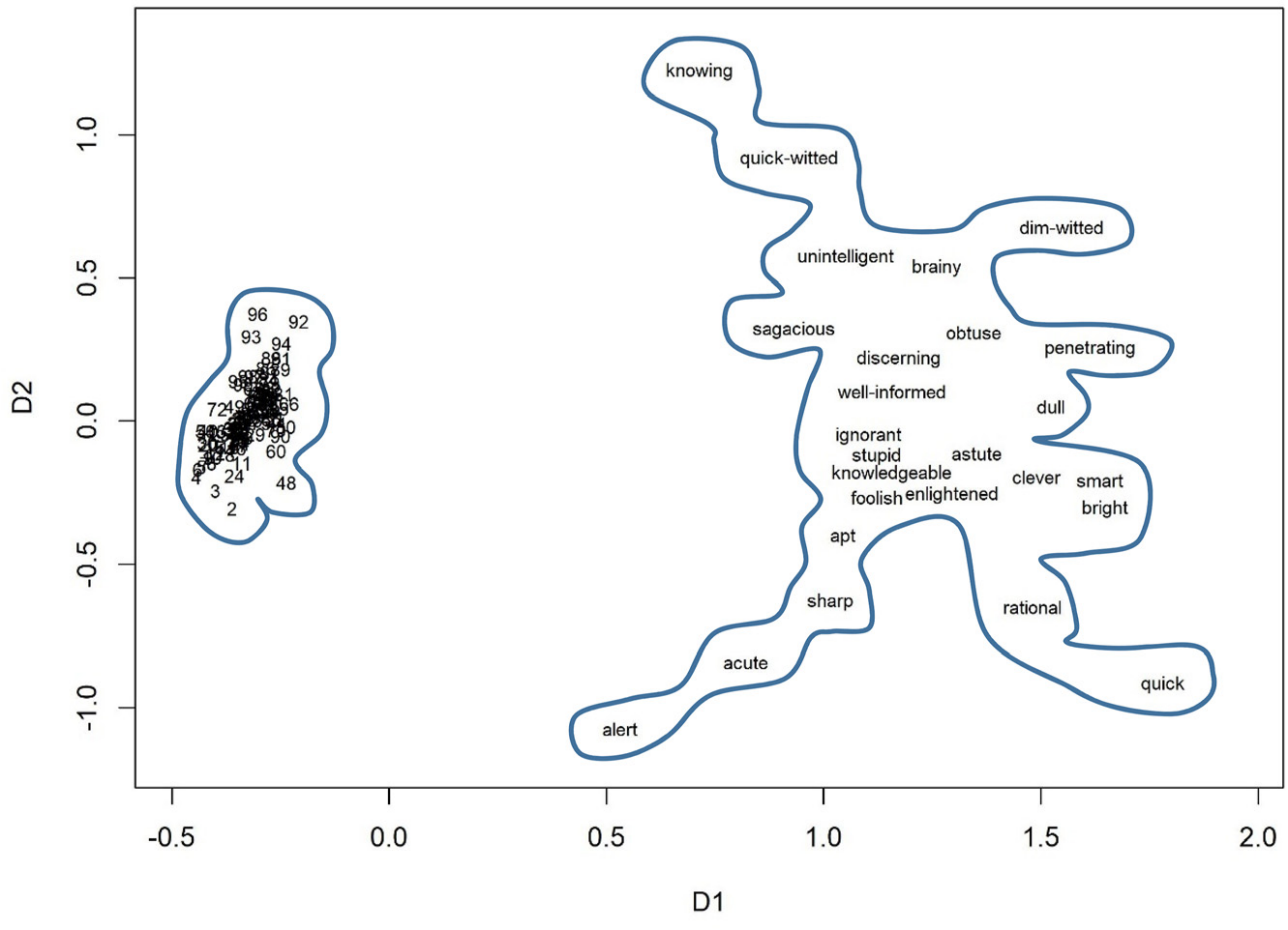
Multidimensional scaling based on *word2vec* similarity measures: numbers and adjectives representing intelligence.

**Figure 3: j_cllt-2023-0105_fig_003:**
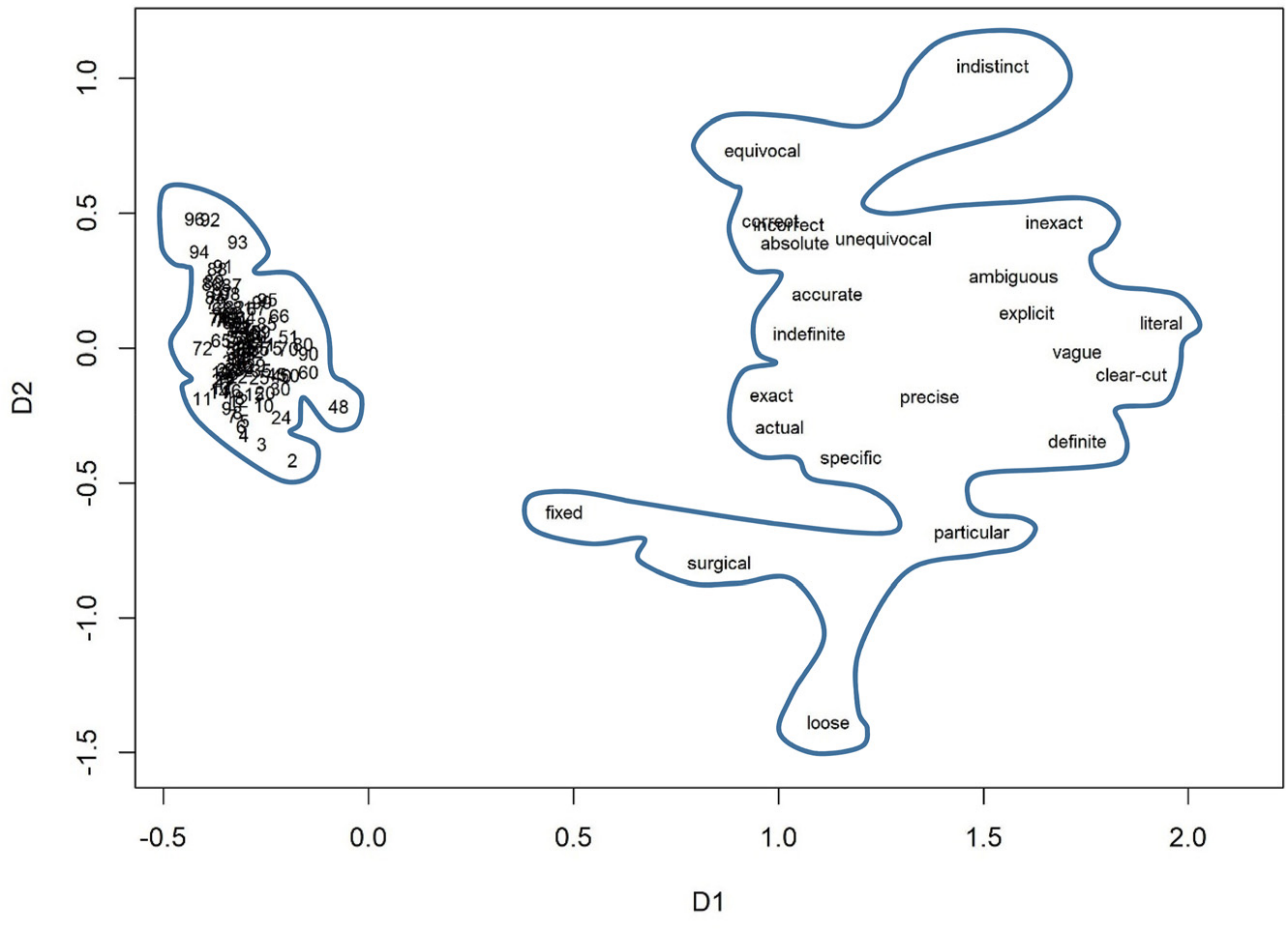
Multidimensional scaling based on *word2vec* similarity measures: numbers and adjectives representing precision.

**Figure 4:**
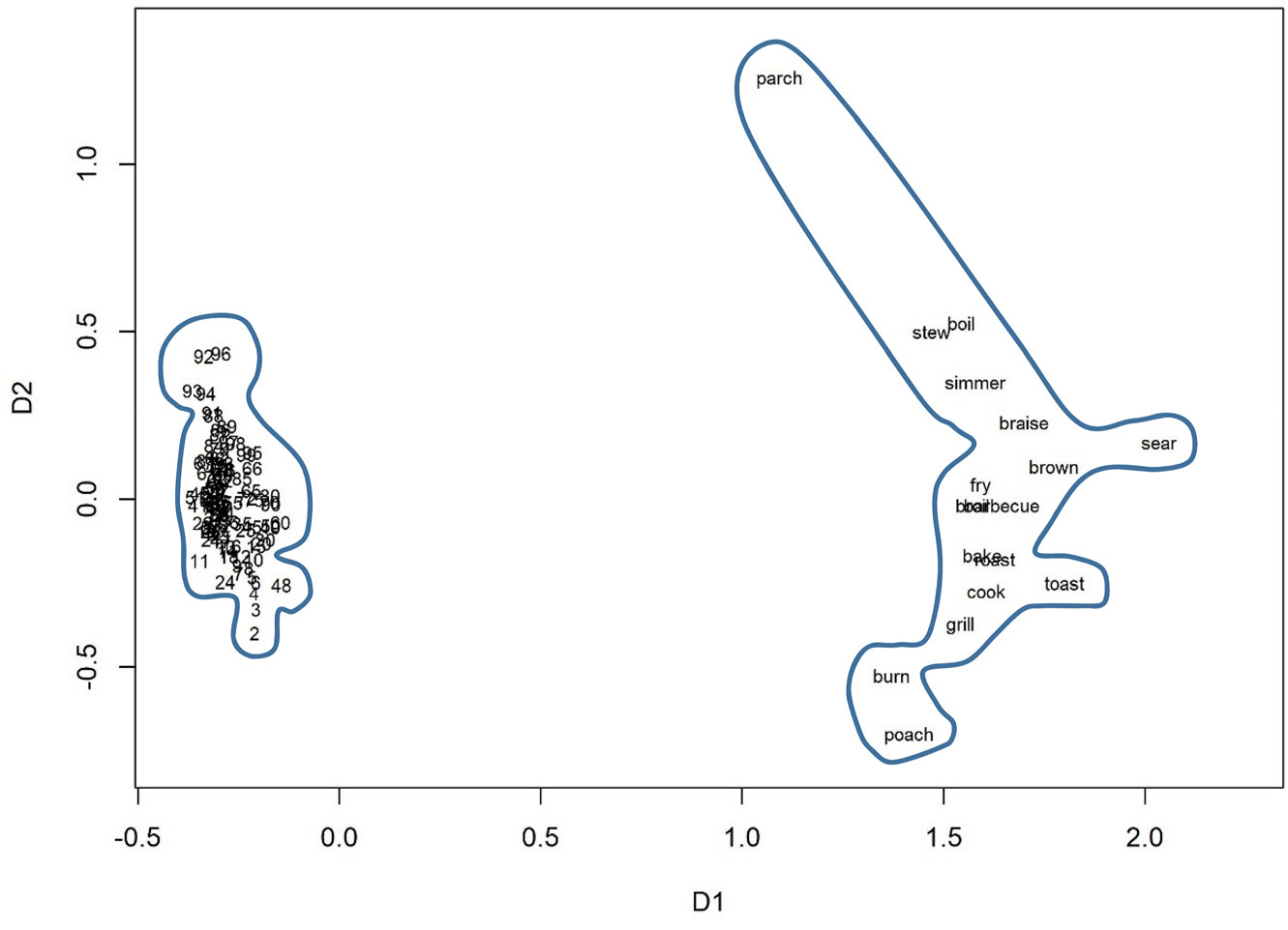
Multidimensional scaling based on *word2vec* similarity measures: numbers and verbs of cooking.

Figure 2 displays the distances between the numerals and between the adjectives representing intelligence. Figure 3 shows the numerals and the adjectives related to precision. Figure 4 displays the numerals and the cooking verbs that were found in the corpus. All three plots demonstrate that the numerals are clustered very densely, while the other domains are sparse. The semantic areas occupied by the sparse-domain lexemes are larger than that of the numbers. This difference is all the more striking given the much larger number of lexemes in the numeral field. In order to control for the number of items in each domain, we computed the average cosine-based similarity between all pairs of lexemes. The average cosine-based similarity between the numbers is 0.78, whereas for the others, the number is much lower: for the verbs of cooking, it is 0.11; for the adjectives describing precision, it is 0.05; and for the adjectives representing intelligence, it is only 0.02.

In sum, Figures 2 [Fig j_cllt-2023-0105_fig_003]–4 show remarkably different distributional patterns for the two types of expressions. The model shows that the number domain is highly densely populated, so that lexemes’ meanings are rather similar to each other, whereas typical lexemes form part of sparse domains, where meanings are relatively different from each other.

We note that the results of this case study have interesting implications for the recent discussions of Large Language Models (LLMs) and their capabilities. Although state-of-the-art LLMs display impressively good results in text generation, their abilities to perform basic arithmetic operations, especially multiplication and division of large numbers, are more modest. Even OpenAI’s most powerful model GPT-4.0 often makes mistakes (Yuan et al. 2023). We believe that the dense semantics of numbers could be one factor responsible for this shortcoming. The contexts in which numbers are used are too alike to allow the algorithm to learn the differences between them and to generate accurate responses.

## 4 Further support for the dense versus sparse lexical domains

Section 4 offers further evidence for the relevance of dense versus sparse domains for language use (Section 4.1), for noncounting communities (Section 4.2), and for language change (Section 4.3).

### 4.1 The sparse shall narrow and the dense shall broaden

We have seen that category operators typically manifest a complementary distribution between numerals and typical lexemes. Thus, selectors *kind of/sort of* overwhelmingly modify typical lexemes (see Sections 2.1 and 2.2 for both English and Hebrew).21Cf. Gries and David (2007). So do *real*(*ly*) (e.g., *real audiophile,* SBC: 016) and *typical*,22An examination of BNC *real/ly 3,5,7,9,18, 20, 25, 100* revealed no such modification for the numerals. In the 9 examples where *real* or *really* did precede a numeral, it was actually the head noun or the whole sentence that was modified, as in *It was a real 100 percent screw up*. and the same is true for selector *typical* in SBC.23Of the 89 *typical* tokens in SBC and LSAC, the one numeral-adjacent *typical* did not modify the numeral (*a typical ten-speed bike*, LSAC). An examination of *typical 2–10* in the BNC revealed 3 similar cases. Moreover, broadeners *around*, *about*, and *approximately* overwhelmingly modify numerals (Ariel 2021). But a complementary distribution account faces two types of counter-examples: (i) the quantitatively marginal, but still exceptional cases noted in Sections 2.1 and 2.2, where numerals were modified by selectors; (ii) the existence of modifiers used for both numerals and typical lexemes.

We here suggest that this almost complementary distribution is not due to selectional restrictions or biases. Rather, it’s the fundamental difference between dense and sparse systems that explains the distribution and interpretation of the category modifiers. Indeed, should a selector modify a numeral, it actually broadens the numeral’s meaning, and when a broadener modifies a typical lexeme, it imposes some selection on it.

Consider selectors first. As expected, selectors *kind of* and *sort of* modifying typical lexemes (e.g., *kinda sore* [SBC: 043]) select nonprototypical interpretations (‘close to sore, but *not* prototypically sore’). But in the rare cases where *sort of* did modify a (measurement) numeral (e.g., *sort of five minutes* [BNC]), its effect was actually to broaden, rather than to select a subset of the denotations. This is why *sort of five* does not exclude ‘exactly 5’. Rather, it creates a broadened range, say ‘4–6’ around the explicit ‘5’. In other words, even if a conventional denotation selector modifies a numeral, it is typically coerced into broadening.

Even the ballpark constructions (e.g., *thousands*), which lie outside the dense number line, are hardly ever narrowed by selectors. There are 56,012 tokens of this construction in the spoken + magazine sections of COCA. Despite the fact that they are definitely associated with multiple denotations, only 12 of them (0.02 %) were modified by a selector. Importantly, in none of these cases did the numeral undergo selection. All 7 *really*s served as ‘truly’, 3/5 *sort/kind of* were not used as numeral modifiers, and most interestingly, two tokens functioned as broadeners rather than selectors:

5.

**Table:** 

There would be **sort of hundreds**, if not thousands, of people around there.

We maintain that when a numeral is (rarely) the target of a selector, the selector turns into a broadener, interpreted as ‘approximately’ (whether the numeral is a round measurement one [6a] or a bona fide nonround count numeral [6b]):

6.

a.

**Table:** 

It was **kinda** a hundred meters away
(www.ontheroadwithrobert.com/p5-costa-rica.html).

b.

**Table:** 

Now, there was **sort of** two things that went on here (LSAC)

Second, most broadeners are restricted to modifying numerals, as in (7), where the denotation is some value within a range constructed around ‘16’:

7.

**Table:** 

They calculate it at a certain percentage, twelve percent, thirteen, sixteen percent **more or less** of that salary level (LSAC)

But *more or less* also modifies typical lexemes. This is a counter-example for a simple complementary distribution account for the modifiers. Note, however, that while the speaker-intended concept conveyed by *sixteen percent more or less* is not in addition selective (it does not exclude the pivot ‘precisely 16’), *decide* in (8a) does exclude a prototypical ‘decision’. The same is true for the ‘knowledge’ involved in (8b):

8.

a.

**Table:** 

We **decide** more or less, we kind of decide we wanted to go to Fosters I guess. (LSAC)

b.

**Table:** 

And you **knew** it more or less, (LSAC)

Indeed, the co-occurring broadener *more or less* and selector *kind of* in 8(a) give rise to the very same ‘nonprototypical decide’ interpretation. In other words, just like selectors, broadeners too involve selection when typical lexemes are involved. Contra Bauer and Huddleston (2002]: 1677), who claim that the suffix –*ish* similarly approximates numerals and typical lexemes, we propose that it only approximates numerals. When modifying typical lexemes –*ish* functions as a selector: *Reddish* (SBC) and *youngish* (LSAC) denote nonprototypical ‘red’/‘young’. But modifying time measuring numerals, such as *oneish* and *thirtyish* (LSAC), it is interpreted as ‘approximately’ (Ariel 2021).

We ran a mini questionnaire in order to support these possibly delicate judgments. We picked the Hebrew ‘more or less’, which is acceptable with both typical lexemes and numerals, and checked whether it functions differently when modifying a typical versus a numeral expression. Thirteen native Hebrew speakers were asked to judge the relative acceptability of the Hebrew counterparts of the following two sentences:

9.

a.

**Table:** 

The color I chose for the living room wall was more or less pink, but I got pink instead.

b.

**Table:** 

I thought there would be more or less thirty people at the party, but there were thirty instead.

We chose a contrastive construction with *but*, and especially with *instead*, in order to show that whereas *more or less* functions as a selector when modifying *pink*, thus excluding ‘central pink’, it functions as a broadener in *more or less thirty*, hence *not* excluding ‘thirty’. If so, “pink” can constitute an alternative to ‘more or less pink’, but ‘thirty’ cannot constitute an alternative to ‘more or less thirty’. Results were quite clear. While many participants commented that both sentences were not perfect,24Indeed, a cooperative speaker would have explicitly marked *pink* as a “central pink” (e.g., *real pink*) here to facilitate the contrast between the two alternatives. a clear majority (11/13) confirmed that (9a) is more acceptable than (9b). Only one participant preferred (9b) (one had no preference). Following his judgment that (9a) is better, one of our participants added the following: “The first one [a] is a bit strange, but one can understand it with a reading that someone had a specific color in mind which is not exactly pink and was disappointed to get “exactly pink” instead. In contrast, in the second sentence [b], there is an actual contradiction in my view, between the two clauses – “30” cannot come **instead of** “more or less thirty” here in my opinion” (R.G. p.c., Dec. 13, 2022, original emphasis). This is exactly our point. It is the difference between dense and sparse systems that accounts for the modifiers’ differential functions. The dense-system numerals can only broaden, whereas sparse-system lexemes necessarily undergo selection.

### 4.2 Numerals in a noncounting community

On our approach, it would seem that numerals should always be construed as members of a dense domain and show no prototype effects. While we claim that this is the overwhelmingly dominant pattern of use, exceptions have been noted in the literature. Interestingly, such cases are restricted to noncounting communities, where specifically the absence of a counting routine has been pointed as the source of the treatment of numerals as approximate (Pica et al. 2004). We take this as further evidence for the inherent connection we propose between a dense domain and precise numerals. The absence of a salient +1 number line in these communities hinders a construal of the numeral domain as dense.25Note, however, that preciseness of interpretation can be inherited from the subitizing use. Indeed, the Mundrukú, a noncounting community, use “one” and “two” precisely (though not “three and onwards”). The crucial point for us, however, is that an entrenched number line based on the successor principle is needed to guarantee a precise interpretation for numerals higher than the subitizing numbers. We thank Bernard Comrie (p.c.) for discussing this point with us.


Flaherty and Senghas (2011), Spaepen et al. (2013), and Spaepen et al. (2011) studied Nicaraguan unschooled homesigners who never learned to recite a counting system, although they live in a numerate culture, and they themselves engage with numbers in connection with money, for example. Here is an example of a participant who was asked to write the Arabic numerals for 1–20 (Flaherty and Senghas 2011):

10.

**Table:** 

1-2-3-4-6-8-7-10-12-11-13-19-16-18-15

Note that quite a few numbers are missing (5, 9, 14, 17, 20), and others are out of order (7, 11, 16, 15). Age-matched hearing participants, equally unschooled, performed this task perfectly. Since they hardly have experience in counting, these signers showed only 27–55 % success rates in ephemeral matching tasks (e.g., where the experimenter taps their knee N times and they have to do the same). Now, if what entrenches the dense domain of the numerals is a practiced counting routine, it is not surprising that these signers treat numerals (other than subitizing ones) as approximate: “They may hold up either 5, 6, or 7 fingers to represent six items” (Spaepen et al. 2013: 497). Their numeral representations are one-to-one mappings between quantities of objects and signs, the latter participating in a typical, sparse lexical domain. This is why, we propose, their number signs do not have rigid boundaries and may be interpreted approximately.

### 4.3 The diachronic stability of numerals

“Most words have about 50 % chance of being replaced by a new noncognate word roughly every 2,000–4,000_y_ (= language years–M.A. and N.L.)” (Pagel et al. 2013: 1). Since words routinely adjust their interpretations in accordance with the context they occur in should some contextual adaptation become highly salient (due to high frequency), it may actually come to serve as the encoded meaning of the expression. Renewal is then necessary in order to encode the original meaning. English *nice* corresponds to Latin “not knowing.” Indeed, once *nice* evolved different meanings, a renewed lexeme *ignorant* was needed.

**Figure 5:**
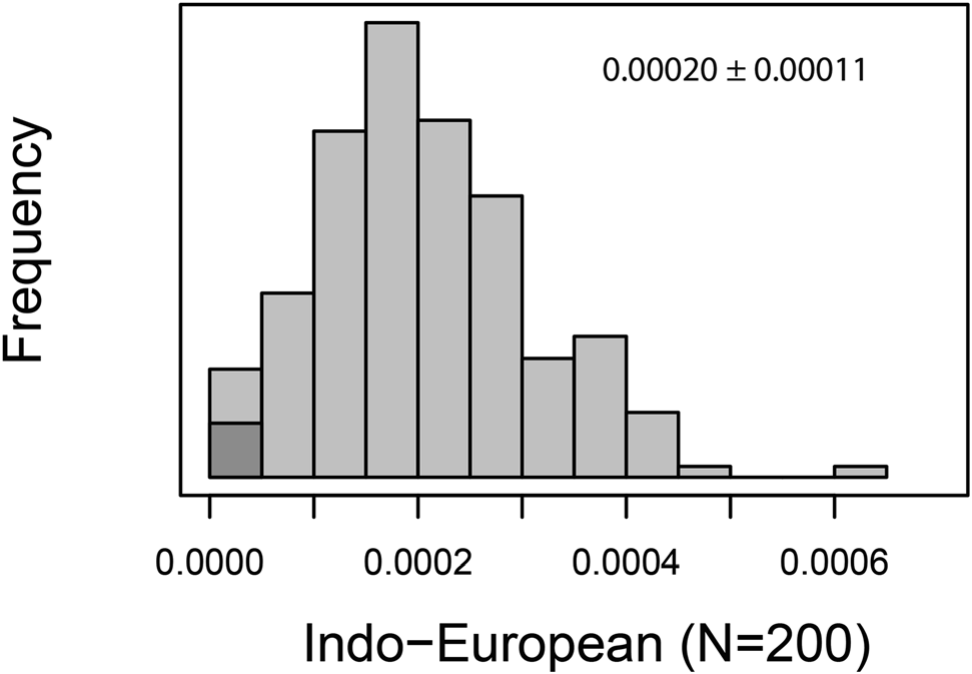
Rates of lexical replacement per annum for Indo-European 1–5 within 200 Swadesh list words. The numerals are in the shaded area. Pagel and Meade (2017) show a similar picture for Bantu and for Austronesian languages, except that specifically ‘one’ is renewed more often than 2–5. Indeed, ‘one’ has other uses than numeral 1.

Numerals, however, “are replaced far more slowly … once every 10,000, 20,000, or even more years” (Pagel et al. 2013: 1). Consider Figure 5, which shows the rate of renewal of Indo-European numerals 1–5, when compared to a basic Swadesh list of 200 words from Pagel and Meade (2017).

We propose that the dense system they are part of explains the absence of synchronic polysemy for numerals, which in turn accounts for their remarkable diachronic stability.26Renewal can also occur due to cultural influence (which is not excluded in Figure 5). But only renewal due to internal semantic change bears on our claim.


## 5 Conclusions

The goal of this paper is to support a distinction between typical, open-class words and numerals with respect to prototype effects, based on their differential distributional patterns. We measured the ratio of broadeners versus selectors for each lexeme type. Typical lexemes are linguistically underspecified. While they naturally broaden, they also need to undergo selection. This is why they are much more often modified by overt selectors than by broadeners. Numerals, on the other hand, have a single, rigid meaning (and denotation), which is why they cannot undergo selection at all, and often need overt modification in order to convey approximate values. We presented quantitative data from English and Hebrew corpora in support of this claim. We then proposed that the source for the different distributional patterns is the different lexical domains that typical lexemes and numerals participate in. Typical lexemes are members of sparse lexical domains, where they each cover a partial region (with multiple denotations), and leave nonlexicalized conceptual spaces between neighboring items. Their borders can then be pushed down to encroach on neighboring meanings. At the same time, the speaker-intended concept may very well be a more specific subset of the potential denotations. Numerals form part of a dense (+1) lexical domain, where the value denoted by the numeral has a single denotation. Hence, narrowing (selection) is impossible. In addition, since the domain is dense and there is no *cognitively salient* unnamed conceptual territory between neighboring members, the numeral cannot broaden its value. An explicit approximator is required for a compositional construction of a range of values.

Indeed, we showed a complementary distribution between numeral operators (broadeners) and typical lexeme operators (selectors). But we also showed that should a literal broadener (e.g., *more or less*) modify a typical lexeme, it necessarily also narrows it, and should a selector (e.g., *sort of*) modify a numeral, it actually broadens it. In other words, it is the lexical domain of the lexeme that determines the function of the operator.

We further supported the distinction between sparse- and dense-domain lexemes with a *word2vec* analysis, which showed strikingly different distributional patterns for numerals versus three typical (sparse-domain) lexemes. Specifically, the model pointed to a high degree of similarity in the use of numerals, as compared to the relatively low similarity in the use of typical lexemes. We tentatively proposed that this may help explain the lower success of OpenAI’s GPT models with arithmetic, but we leave this topic for future research. Next, the lower rate of precise interpretations for nonround numerals among the deaf community in Nicaragua supported our claim about the association between a salient number line and precise interpretations. Finally, the remarkable diachronic stability of numeral meanings supports our analysis too: the dense domain explains the low contextual polysemy, which in turn accounts for the low rate of semantic change.

But, then, one can ask why it is that we have sparse versus dense lexical domains in the first place. Clearly, the linguistic norm is lexical underspecification building on a sparse system. Modification of typical lexemes is then motivated by the fact that the speaker’s message is (usually) relatively specific (e.g., that the denotation is more or is less prototypical), but the encoding options provided by a sparse lexical domain are not rich enough. The typical situation with numerals is the opposite. The speaker may very well wish to communicate an *imprecise* message (she may only have an approximate range in mind), but the lexical domain only offers precise alternatives. It is this “overspecificity” of the dense lexical domain that makes the numerals linguistically exceptional. While there are many linguistic strategies (such as modification, dedicated constructions, personality numbers) for “overcoming” the numeral system’s overspecificity, the dense numeral system itself nonetheless lives on. We conclude that our cultural numeracy, based on the successor principle responsible for the dense number system, constitutes a powerful expressive force in communication.

## References

[j_cllt-2023-0105_ref_001] Ariel Mira (2004). Most. *Language*.

[j_cllt-2023-0105_ref_002] Ariel Mira, von Heusinger Klaus, Turner Ken (2006). A ‘just that’ lexical meaning for *most*. *Where semantics meets pragmatics*.

[j_cllt-2023-0105_ref_003] Ariel Mira, Mauri Caterina, Fiorentini Ilaria, Goria Eugenio (2021). Why it’s hard to construct ad hoc number concepts. *Building categories in interaction: Linguistic resources at work*.

[j_cllt-2023-0105_ref_004] Ariel Mira, Levshina Natalia (2021). Round and measurement numerals are precise by default. Work report. ..

[j_cllt-2023-0105_ref_005] Armstrong Sharon, Gleitman Lila R., Henry Gleitman (1983). What concepts might not be. *Cognition*.

[j_cllt-2023-0105_ref_006] Bauer Laurie, Huddleston Rodney, Huddleston Rodney, Pullum Geoffrey (2002). Lexical word formation. *The Cambridge grammar of the English Language*.

[j_cllt-2023-0105_ref_007] Biber Douglas (1995). *Dimensions of register variation: A cross-linguistic comparison*.

[j_cllt-2023-0105_ref_008] Breheny Richard (2008). A new look at the semantics and pragmatics of numerically quantified noun phrases. *Journal of Semantics*.

[j_cllt-2023-0105_ref_009] Bultinck Bert (2005). *Numerous meanings. The meaning of English Cardinals and the Legacy of Paul Grice*.

[j_cllt-2023-0105_ref_010] Bybee Joan L. (2006). From usage to grammar: The mind’s response to repetition. *Language*.

[j_cllt-2023-0105_ref_011] Carston Robyn, Carston Robyn, Uchida Seiji (1998). Informativeness, relevance and scalar implicature. *Relevance theory: Applications and implications*.

[j_cllt-2023-0105_ref_012] Carston Robyn (2002). *Thoughts and utterances: The pragmatics of explicit communication*.

[j_cllt-2023-0105_ref_013] Casati Roberto, Reboul Anne (2014). Numerals and word sequences. *Mind, values, and metaphysics: Philosophical essays in honor of Kevin Mulligan*.

[j_cllt-2023-0105_ref_014] Chierchia Gennaro, McConnell-Ginet Sally (1990). *Meaning and grammar: An introduction to semantics*.

[j_cllt-2023-0105_ref_015] Comrie Bernard (2022). The arithmetic of natural language: Toward a typology of numeral systems. *Macrolinguistics*.

[j_cllt-2023-0105_ref_016] Culbertson Jennifer, Schouwstra Marieke, Kirby Simon (2020). From the world to word order: Deriving biases in noun phrase order from statistical properties of the world. *Language*.

[j_cllt-2023-0105_ref_1040] Cummins Chris (2015). *Constraints on numerical expressions*.

[j_cllt-2023-0105_ref_017] Dehaene Stanislas (2011). *The number sense*.

[j_cllt-2023-0105_ref_018] Du Bois John W., Chafe Wallace L., Meyer Charles, Thompson Sandra A., Englebretson Robert, Martey Nii (2000–2005). *Santa Barbara corpus of spoken American English, Parts 1–4*.

[j_cllt-2023-0105_ref_1041] Firth John R. (1957). *Studies in linguistic analysis: Special volume of the Philological Society*.

[j_cllt-2023-0105_ref_019] Flaherty Molly, Senghas Ann (2011). Numerosity and number signs in deaf Nicaraguan adults. *Cognition*.

[j_cllt-2023-0105_ref_020] Geeraerts Dirk (2010). *Theories of lexical semantics*.

[j_cllt-2023-0105_ref_021] Geurts Bart, Vogeleer Svetlana, Tasmowski Liliane (2006). Take “five”: The meaning and use of a number word. *Non-definiteness and plurality*.

[j_cllt-2023-0105_ref_047] Gries Stefan Th., David Caroline V., Pahta Päivi, Taavitsainen Irma, Nevalainen Terttu, Tyrkkoö Jukka (2007). *Studies in variation, contact and change in English. Volume 2. Towards multimedia in corpus studies.*.

[j_cllt-2023-0105_ref_022] Horn Laurence R. (1989). *A natural history of negation*.

[j_cllt-2023-0105_ref_023] Kadmon Nirit (2001). *Formal pragmatics*.

[j_cllt-2023-0105_ref_024] Katzir Nicole, Levshina Natalia, Ariel Mira (2024). Rethinking approximation in round numbers. ..

[j_cllt-2023-0105_ref_025] Koenig Jean-Pierre (1991). Scalar predicates and negation: Punctual semantics and interval interpretations. *Proceedings of Chicago Linguistic Society 27*.

[j_cllt-2023-0105_ref_1048] Krifka Manfred, Restle David, Zaefferer Dietmar (2002). *Sounds and systems. Studies in structure and change. A Festschrift for Theo Vennemann*.

[j_cllt-2023-0105_ref_026] Lakoff George (1987). *Women, fire, and dangerous things: What categories reveal about the mind*.

[j_cllt-2023-0105_ref_027] Langacker Ronald W. (1987). *Foundations of cognitive grammar. Vol. 1: Theoretical prerequisites*.

[j_cllt-2023-0105_ref_028] Lehrer Adrienne (1974). *Semantic fields and lexical structure*.

[j_cllt-2023-0105_ref_1043] Levshina Natalia, Heylen Kris, Boogaart Ronny, Colleman Timothy, Rutten Gijsbert (2014). *Extending the scope of construction grammar*.

[j_cllt-2023-0105_ref_029] Lyons John (1977). *Semantics*.

[j_cllt-2023-0105_ref_1044] Mikolov Tomas, Chen Kai, Corrado Greg (2013). Efficient estimation of word representations in vector space. ..

[j_cllt-2023-0105_ref_030] Murphy Gregory L. (2004). *The Big Book of concepts*.

[j_cllt-2023-0105_ref_1046] Pagel Mark, Atkinson Quentin, Calude Andreea, Meade Andrew (2013). Ultraconserved words point to deep language ancestry across Eurasia. *PNAS*.

[j_cllt-2023-0105_ref_1049] Pagel Mark, Meade Andrew (2017). The deep history of the number words. *Philosophical Transactions of the Royal Society of London B*.

[j_cllt-2023-0105_ref_031] Pica Pierre, Lemer Cathy, Izard Veronique, Dehaene Stanislas (2004). Exact and approximate arithmetic in an Amazonian Indigene Group. *Science*.

[j_cllt-2023-0105_ref_032] Spaepen Elizabet, Coppola Marie, Flaherty Molly, Spelke Elizabeth (2013). Generating a lexicon without a language model: Do words for number count?. *Journal of Memory and Language*.

[j_cllt-2023-0105_ref_033] Spaepen Elizabet, Coppola Marie, Spelke Elizabeth S., Carey Susan E., Goldin-Meadow Susan (2011). Number without a language model. *Proceedings of the National Academy of Sciences*.

[j_cllt-2023-0105_ref_034] Trier Jost (1934). Das sprachliche Feld. Eine Auseinandersetzung. *Neue Jahrbücher für Wissenschaft und Jugendbildung*.

[j_cllt-2023-0105_ref_035] Ullmann Stephen (1967). *The principles of semantics*.

[j_cllt-2023-0105_ref_036] van Tiel Bob, Franke Michael, Sauerland Uli (2021). Probabilistic pragmatics explains gradience and focality in natural language quantification. *Proceedings of the National Academy of Sciences*.

[j_cllt-2023-0105_ref_037] Wilson Deirdre, Carston Robyn, Burton-Roberts Noel (2007). A unitary approach to lexical pragmatics: Relevance, inference and ad hoc concepts. *Pragmatics*.

[j_cllt-2023-0105_ref_038] Woodin Greg, Winter Bodo, Littlemore Jeannette, Perlman Marcus, Grieve Jack (2023). Large-scale patterns of number use in spoken and written English. *Corpus Linguistics and Linguistic Theory*.

[j_cllt-2023-0105_ref_039] Wynn Karen (1992). Children’s acquisition of the number words and the counting system. *Cognitive Psychology*.

[j_cllt-2023-0105_ref_1045] Yuan Zhen, Yuan Hongyi, Tan Chuanqi, Wang Wei (2023). How well do Large Language Models perform in arithmetic tasks?. ..

